# Professor David Philippe Walther (1909-1974) and the development of orthodontics at the Royal Dental Hospital of London

**DOI:** 10.1038/s41415-025-8403-2

**Published:** 2025-08-22

**Authors:** Stanley Gelbier

**Affiliations:** Honorary Professor and Head of the Unit for the History of Dentistry, Faculty of Dentistry, Oral and Craniofacial Sciences, King´s College London, Guy´s Hospital, Tooley Street, London, SE1 1UL, United Kingdom

## Abstract

Professor Philippe Walther was a major player in the development of orthodontics in the United Kingdom in the post-war years, especially at the postgraduate level. He and colleagues at the Royal Dental Hospital of London (RDH) and the Eastman built on existing service and education programmes to develop a highly successful one-year postgraduate course at the RDH, leading to the Diploma in Orthodontics exam of the Royal College of Surgeons of England. He also helped to build a major unit at Great Ormond Street Hospital for Sick Children.

## Key points

- Orthodontics hardly existed as a specialty in the UK until after World War II. The establishment of the NHS provided the environment for both demand and accessibility for dentistry, including orthodontics.
- As always, the development of this specialty needed people to drive its acceptance and establishment. This paper highlights one such person, amongst several.
- The relative physical closeness allowed consultants and budding specialists to go back and forth between two major hospitals - one paediatric, the other dental - which helped to advance the specialty and benefit the patients.

## David Philippe Walther

Professor David Philippe Walther (generally called Philip, but on occasion signing as David) was born on 18 September 1909 in St Leonards-on-Sea, Sussex, to David Rodolphe Philippe (a physician) and Miriam Dora (*née* Mason) Walther.^1^ His siblings were John Rodolphe (1905), Alfred Leslie (1911) and Evelyn Malcolm Echalaz (1913).

His primary schooling was at St Edward's School in Oxford, founded in 1863 by the Reverend Thomas Chamberlain. The latter was senior student and honorary canon of Christ Church Oxford, as well as the vicar at St Thomas the Martyr in Oxford. It originally intended to primarily educate the sons of middle-class clergy and to emphasise the teachings of the Anglican faith as its core priority. He then went to Norwich Grammar School.

On 31 January 1930, Philip was registered on the General Medical Council's Register of Medical and Dental Students. He then entered Guy's Hospital Medical and Dental School to study dentistry, gaining the LDSEng in 1934.

**Fig. 1:**
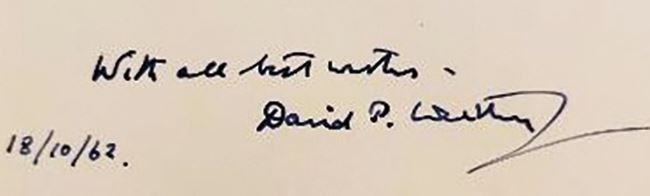
Signature of David P. Walther

Although known to his friends as Philip, in 1962 he signed a copy of his ‘notes' for a student as David P. Walther (Fig. 1).

## Dental career

After qualification, Philip Walther was appointed to a house surgeon post in the Department of Children's Dentistry at Guy's Hospital, which included surgery and orthodontics, remaining there for one year. At the same time, he undertook private practice in Hampstead in North West London, largely devoted to children's dentistry and orthodontics. Whilst there, Walther must have heard of Gwynne Evans' and Clifford Ballard's groundbreaking work at the Upper Respiratory Clinic at St Bartholomew's Hospital, which may have increased his interest in orthodontics. He joined the British Society for the Study of Orthodontics (BSSO) in 1947.

He continued to practise part-time but returned to Guy's to study medicine. Despite the arrival of World War II, Philip maintained his medical studies, gaining the MRCS LRCP in 1941. He then undertook house physician and house surgeon posts at the Miller General Hospital in Greenwich. After enlistment as a captain in the Royal Army Medical Corps, Philip served as a doctor on a hospital ship between the United Kingdom (UK) and North Africa. He landed in France on D-Day with one of the first medical teams, serving in field ambulances, casualty clearing stations and general hospitals in France, Holland and Germany, and at some concentration camps.^2^

Returning from the Army, he became a registrar in the Department for Children and Orthodontics at Guy's, holding that post for two years, developing a major interest in orthodontics.

Next, Walther was a half-time senior orthodontic registrar at Great Ormond Street Hospital for Sick Children (GOS) (1948-1950). He was also a senior hospital dental officer and orthodontist at the Eastman Dental Hospital (1949-1954). By then, much was going on in UK orthodontics.

## Early orthodontic history at the Royal Dental Hospital

An 1882 ‘student supplement' to the *British Journal of Dental Science* stated that lectures on irregularities of teeth were given during dental surgery and pathology courses at the Dental Hospital of London (forerunner of RDH) and the National Dental Hospital.^3^ A formal course of lectures on what became ‘orthodontics' was delivered at Guy's by dental surgeon, John Henry Badcock (LDS 1887; MRCS LRCP 1890; FRCS 1932) (1864-1953) in 1900.

Orthodontics began in the Children Department's ‘regulation room' at the Royal from 1905, with its own house surgeon; that it entailed necessary treatment rather than training for students was recognised by the Committee of Management.^4^ It was therefore decided that the hospital rather than the school should pay the salary of the house surgeon. In that year, it was decided not to make charges for treatment in the Children's Department. However, by 1919, an income of £10.85 was reported for supplying regulating appliances. As school clinics sprang up in London, their work brought to RDH many children needing orthodontic treatment. Its Medical Committee wrote to the Board of Education about the issue, pointing to problems for school dentists: a lack of time plus difficulty of diagnosis, treatment and manufacture of appliances. They suggested the Hospital could assist by arranging for members of staff to advise on cases sent to them for the purpose. From 1919, it accepted children from clinics in Ealing, West London. The Borough Council paid the RDH 7 shillings and 6 pence per child, plus an annual donation of 10 guineas to the hospital funds. Under that arrangement, which continued for five years, orthodontic cases flowed into the Hospital. Something had to be done for their treatment. For training purposes, up to five qualified dentists attended for one or two sessions a week for three months, paying for their instruction while carrying out treatment under the supervision of a member of the senior staff. To meet the demand for appliances, a full-time technician was employed.

By June 1925, there was a waiting list of 200 children. The arrangement was like one in force during the 1960s and 1970s. At that time, practitioners were enrolled by the school as half-time orthodontic postgraduates and simultaneously appointed by the hospital as half-time NHS (National Health Service) orthodontic registrars. In spite of that treatment, the amount of orthodontic education for undergraduates remained negligible until 1927. It was included in the lecture course on ‘dental disease in children' by F. St Jermain (known as St John) Steadman (LDS 1902; MRCS 1907; DPH 1911) (1880-1943). In 1927, the Medical Committee decided a course of demonstrations in orthodontics should be given during the six weeks preceding the final LDS examination.

Major developments began in 1930 when Miss Kathleen Corisande Smyth (LDS 1923) (1902-1953) was appointed as demonstrator in orthodontics with funding from the Dental Board of the UK. In 1931 came a full course of lectures in orthodontics. In that year, (later Sir) Norman Bennett (LDS 1894; BCh 1896; MB Cantab 1900) (1922-2005) was appointed as the first lecturer in orthodontics. By the year-end, plans were underway for the establishment of a department of orthodontics.

An important postgraduate national orthodontic development came in 1949. Recognising a need for proper training, the Faculty of Physicians and Surgeons of Glasgow established a Diploma of Dental Orthopaedics (DDO). It was a further five years before the Royal College of Surgeons of England established its Diploma in Orthodontics.

A major academic development for RDH came in 1951, when the University of London appointed Miss Corisande Smyth as the first UK reader in orthodontics, tenable at the London School of Dental Surgery of the Royal Dental Hospital. By the time Walther arrived, Smythe and Bennett had the undergraduate teaching of orthodontics well-organised. In 1956, Clifford Ballard (LDS 1934; MRCS LRCP 1940; DDO 1954) (1910-1997) became the first professor at the Eastman Institute of Dental Surgery. He had worked under Smyth at the Royal for 12 years (1940-1952) while learning his craft.

The NHS was gradually recruiting many more consultants in virtually every branch of medicine and dentistry, including orthodontics. At the same time, the universities became more involved in orthodontic education. The scene was set for Philip's advancement.

## Walther's senior posts

A consultantship in orthodontics at the Eastman and GOS came for Philip in 1954. Within two years, he had the Diploma in Orthodontics and was appointed by the University as reader in orthodontics at RDH on Smythe's death and became director of its orthodontic department. He developed large and enthusiastic orthodontic units at both RDH and GOS and retained his link with GOS. The year 1961 saw Walther's promotion to professor. By then, he was also honorary consultant to the St George's Hospital Group.

All the while, he continued research projects. As a result, in 1964, he gained an MDS degree from the University of London. The FDSRCS followed in 1966.

The undergraduate and postgraduate teaching programmes at the Royal expanded greatly under his guidance. The development of a one-year postgraduate orthodontic course at the Royal was a joint effort with the Eastman, led by Clifford Ballard, who, for many years, gave lectures to the Royal's postgraduates. The strong postgraduate presence in turn benefited the undergraduates (including the author). Walther's obituary suggests it was his commitment to the postgraduate training of both hospital and academic staff plus dentists on the one-year courses which were perhaps his greatest contributions to dentistry for which he will be remembered. The link with GOS was maintained by several clinicians and greatly benefited the postgraduate training and research programmes.

Walther was particularly concerned with patients afflicted by cleft lip and palate problems, but he retained an interest in the general health of children's teeth beyond orthodontics. In spite of his interest in treatment of cleft lip and palate, he only published one paper on this subject; indeed, he only published five papers in British journals, the first as a second author to Ballard. It is noteworthy that, in an important 1956 Dental Board publication, ‘The aetiology of irregularity and malocclusion of the teeth', there are references to the works of Norman Bennett (four papers), Corisande Smyth (three papers) and Clifford Ballard (six papers), but none to Walther.

While discussing the orthodontic department, mention should be made of John Hovell (1910-1988)^5^ who played a significant role. He was, uniquely, an NHS consultant in both orthodontics and oral surgery. When the Department of Health enquired how many orthodontic senior registrars were needed at RDH, Hovell, being ex-service and aware of how the civil service behaved, advised they should ask for twice the number they needed. The hospital was astonished when they got all four posts requested, which allowed four additional sessions of undergraduate teaching. The opportunity for career advancement came for those registrars completing the Royal's postgraduate course as the hospital's senior registrars obtained consultant posts.

His obituary confirmed that Philip Walther was a quiet person who did not enjoy public speaking. Walther diligently served on a number of university, hospital and society committees, always well-prepared, with strong principles. A major contribution was his sustained and very valuable contribution as secretary of the European Orthodontic Society from 1955 until he died. Under him, it grew from a small band of enthusiasts to a very large membership, with many worldwide corresponding members. Walther was President of the BSSO in 1960.

## Publications

In spite of Walther's interest in the treatment of clefts, he only he only authored one paper on this subject. Indeed, unusually for an academic, he only published five papers, the first co-authored with Ballard. His most significant was his 1960 East Anglian study,^6^ based on his MDS research. This compares with Ballard's 15 papers and Bill Houston's (his senior lecturer) 40 publications.

**Fig. 2:**
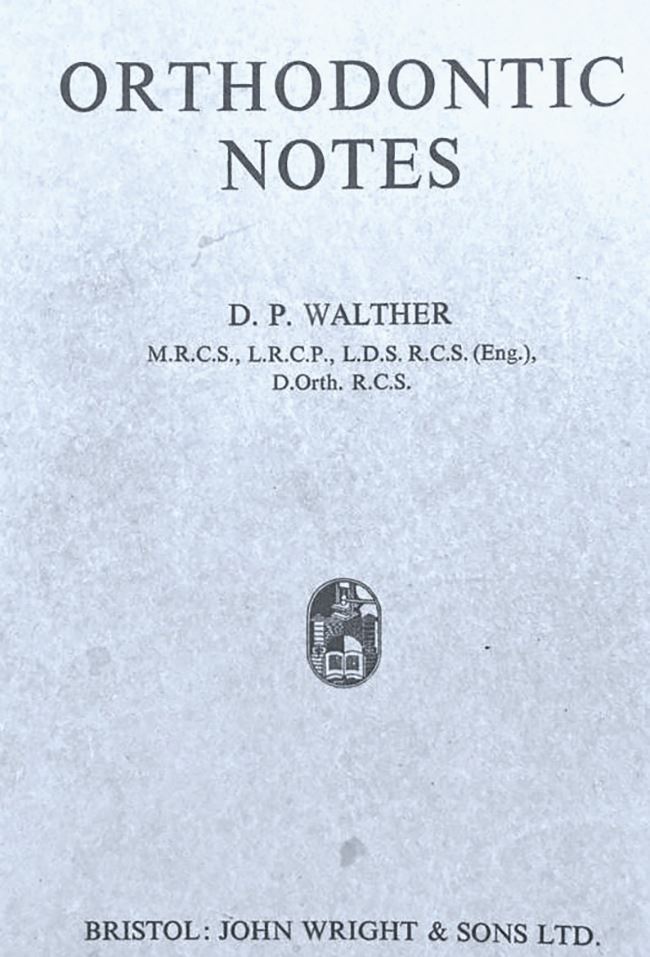
Front cover of Orthodontic Notes

In 1960, John Wright & Sons of Bristol published Walther's *Orthodontic Notes* (Fig. 2), which became widely read and loved by undergraduates (including the author). Professor Clifford Ballard wrote in a foreword:

- ‘Because orthodontics is still regarded as a subject of little importance in undergraduate teaching, and one to which only superficial reference has to be made, the importance to all branches of dentistry of the fundamental biological concepts expressed in these notes may not be appreciated for some time. They are the link between anatomy and physiology and clinical practice. These notes, therefore, are not just another contribution to the subject of orthodontics; they are a step towards making dentistry a branch of applied human biology'.

It went on to have five editions.

Walther followed in 1966 by editing *Current Orthodontics*, with contributions from eight other teachers. In 1983, W. J. B. Houston updated the *Orthodontic Notes* to *Walther's Orthodontic Notes* (Bristol, London and Boston: Wright PSG), which, in 1994, became *Walther and Houston's Orthodontic Notes*.

## Walther's personal life

Philippe Walther married Barbara (Bobbie) Brook (1918-2003) on the day war was declared in 1939, in Battle, Sussex; he aged 30, she 21 years. They set up home at The Granary, Bramley in Guildford, Surrey, he having previously lived in Hampstead. By then, he was a qualified dentist but still a medical student. They later had two children, son, Robert and daughter, Jane.

Philip enjoyed gardening. He also gained much pleasure from countryside pursuits, especially associated with life on a farm. As a result, he spent much of his free time out of London. He had a love of animals and was often to be seen with his beloved dog. He enjoyed the life of a country gentleman and managed in his later years to negotiate an arrangement with the University of London whereby he used his annual leave as half days on Mondays and Fridays enabling him to spend long weekends at his property in Bridgnorth, Shropshire throughout the year.^7^ Walther eventually retired to Cantreyn in Bridgnorth, Shropshire.

Walther was primarily an urbane and charming gentleman who everybody liked and who hated conflict. This enabled him to attract and build a talented team. He disliked public speaking and public debate. Thus, he gave few lectures to the BSSO and seldom, if ever, contributed to the discussion of papers presented by others as recorded in the BSSO Transactions. He seems to have made little input to the running of RDH. Thus, while the contributions made by senior members of the Royal's Orthodontic Department are described in its history by Smith and Cottell, Walther receives no mention.

David Phillippe Walther died on 31 December 1973.

## The Philip Walther Prize

Upon his death, the RDH established a Philip Walther Prize for the student attaining the highest mark in the MSc in Orthodontics exam: £50 plus a RDH shield.^8^ The prize is continued by the successor school at King's College London (KCL). For example, in 2017, Xavier Guilherme received the prize from KCL's Centre for Craniofacial and Regenerative Biology for attaining the highest grades in the University of London MSc examination.
